# Telephone, video, equity and access in virtual care

**DOI:** 10.1038/s41746-021-00528-y

**Published:** 2021-11-18

**Authors:** Tyla Thomas-Jacques, Trevor Jamieson, James Shaw

**Affiliations:** 1 Institute for Health System Solutions and Virtual Care, Women’s College Hospital, University of Toronto, Toronto, Canada; 2 Department of Medicine, Unity Health Toronto, Toronto, Canada; 3 Joint Centre for Bioethics, University of Toronto, Toronto, Canada

**Keywords:** Health policy, Health services, Technology

## Abstract

Current public health measures catalyzed a large shift to virtual care, resulting in a great uptake in telephone and video-enabled care. While pre-pandemic public healthcare funding rarely covered the telephone as a reimbursable care delivery model, it has proven a crucial offering for many populations. As the new standard of virtual service delivery is being solidified, simple technological solutions that provide access to care must continue to be supported. This paper explores an important consequence of relying on complex technologies as the new standard of virtual care: the risk of exacerbating health disparities by enabling a deeper digital divide for marginalized populations.

The COVID-19 pandemic introduced a mandate to restrict in-person contact between providers and patients, giving rise to a dramatic increase in the use of virtual care for ambulatory services^1^. Among the various issues raised by this shift to virtual care is the challenge of ensuring health equity when delivering care. The growth in the use of video visits, for example, poses obvious challenges for patients without access to technology, those with low digital literacy, and those without a private space for video interaction (such as in smaller, multi-tenant dwellings)^2^. These realities faced by some patients represent the digital divide in health care, which has been exacerbated by measures to reduce in-person contact during the COVID-19 pandemic^3^.

One important point needs to be clearly stated as providers get used to the new normal of virtual care: when health systems focus on more technically advanced but complicated solutions as their default modes of care, they are more likely to enhance disparities in access to and outcomes of health care. In contrast, when health systems support the technically simplest solution required to solve any given problem, they both generate a solution that works for the largest proportion of people and reduce costs overall.

In this Comment, we provide a rationale for discovering and deploying technologically simple solutions for the sake of inclusive population health in the short term, while investing in more advanced virtual care where appropriate in the long term. We acknowledge an important tension at the core of our argument between access and quality of care, and we explore the trade-offs between access and quality after presenting the case for technologically simple solutions.

New technologies and innovations can improve the productivity of health care systems, yet at the same time technologies can exacerbate disparities between patient groups^4^. This is because the benefits of digital technologies accrue to the people who have regular access to those technologies, including their use for activities related to the management of health and health care^5,6^. The digital divide clarifies that the challenge is not only about access to the Internet and connected devices, but also about the motivation and digital literacy to use those technologies in meaningful ways^6^. More complex technologies certainly have no immediate benefit to patients who cannot access them at all, but they also introduce a much higher baseline for digital literacy in order to leverage them optimally for health-related reasons even for those with access^5^. This acknowledgement of the implications of the digital divide for meaningful engagement with virtual care sets the context for our discussion of telephone and video visits.

## Telephone and video visits

The COVID-19 pandemic has demonstrated the challenge of the digital divide for health systems through experiences of care with two technologies: video and the conventional telephone. Prior to the pandemic, funding options for virtual care were quite narrow and based on the medium of care delivery^7^; for example, providers might be reimbursed for video visits but not for phone visits^7^. Since the onset of the pandemic, health care payers have extended virtual care funding, often on an emergency basis, to include more media, including both telephone and video over a wider variety of platforms^7^.

This extended funding model was the case in Ontario, Canada, where services deemed medically necessary are publicly covered by a single government payer, and privately delivered by providers. Prior to COVID-19 only video visits were a reimbursable service under Ontario provincial public insurance, and then only through a single technological provider^8^. Similarly, in the United States, interim regulatory changes have also expanded what qualifies as a reimbursable telehealth service outside of previous definitions to include telephone visits for Medicare and Medicaid^9^. These policy changes generated a unique opportunity to more clearly understand the demographic profiles of patients engaging with virtual care using the telephone versus the more advanced technologies that enable video visits. Research published on this topic in the context of COVID-19 provides important insights into the equity implications of reimbursing and relying upon these different modalities of virtual care.

Cohort studies completed by Eberly et al.^10^, Schifeling et al.^11^, and Rodriguez et al.^12^ during the COVID-19 pandemic separately examined the patient characteristics associated with the use of telephone versus video visits in various health systems in the United States. These three studies share a common finding that patients who are Black and living in lower-income areas were more likely to engage in telephone visits, and patients who are white and from higher-income areas were more likely to engage in video visits. Precise rates of usage varied and these studies present additional findings beyond those summarized here. However, this overarching observation illustrates the point that over time these patterns of usage would reinforce and likely exacerbate existing inequities in access to and outcomes of health care along the lines of race and income associated specifically with virtual care.

In commenting on their finding that clinicians and practices contributed to driving decisions regarding virtual care modality (telephone or video), Rodriguez et al.^12^ point out that practices serving “primarily underserved patients may be less equipped to provide video visits given additional implementation requirements”. These remarks illustrate an important point that ought to accompany the observation of disparities in the use of video visits, which is that health care providers have varying degrees of control over the modality through which they will deliver care. At times, circumstances of the patient, clinician, or practice setting prevent the possibility of using video visits, such as the lack of access to video-enabled technology by the patient or the clinician. However, there are circumstances where clinicians do have influence over whether telephone or video visit modality is chosen for a given encounter, and these scenarios raise the importance of potential differences in quality between the two modalities.

Research by Thomson et al.^13^ surveyed physicians in a single health system in the United States to examine physician perspectives on the appropriate mix of virtual and in-person services following the COVID-19 pandemic, and perceptions of quality of those services. These authors identified variation between the 51 medical specialties included in the survey but found that a majority of respondents were satisfied or very satisfied with the care they were able to provide via video (78%). A 2018 systematic review including eight articles comparing the quality of video visits versus telephone visits modestly supports this viewpoint, suggesting that evidence indicates that video visits are comparable and at times superior in effectiveness to telephone visits^14^, albeit across a very limited sampling of use cases that cannot easily be generalized to medicine writ large. Considerable further research is necessary on this topic to establish this point with confidence.

## Access and quality

The apparent preference for video visits among physicians reported in Thomson et al.^13^ and the possible advantage in effectiveness of video over telephone visits reported in Rush et al.^14^ in some clinical situations raise the crucial issue of the trade-off between access and quality of care. Where the telephone is offered and reimbursed as a modality of virtual care, essentially everyone can enjoy access to care of meaningful clinical quality. Where video visits are offered and reimbursed as an exclusive modality of virtual care, only those in more privileged positions will enjoy access to care that, in certain situations, may be of higher quality. What does this tension suggest about reasonable next steps for virtual care?

We answer this question by returning to our primary contention in this Comment: that health systems ought to support technologically simple solutions for the sake of inclusive population health in the short term, while investing in more advanced virtual care where appropriate in the long term. Keeping the telephone reimbursable and available as a modality of care delivery for the foreseeable future will ensure that those without access to video visit technology can continue to receive virtual care that is convenient and timely—and there is no strong evidence that care by telephone can be universally categorized as “unsafe”, justifying its removal. Of course, the telephone is not appropriate for every clinical need. To account for this, future work should establish a clinical decision process to clearly indicate the clinical use cases in which phone is most appropriate, of moderate utility, or presents a risk if used. The same scrutiny should be equally applied to video, which itself has not been established as universally appropriate or safe, and also to more novel applications of virtual care like secure text messaging or remote patient monitoring. However, the absence of such a framework is not a reason to halt the reimbursement of telephone visits, but rather a reason to expedite the development of a clearer strategy to promote health equity in virtual care.

The first clause of our primary contention in this paper implies that it is important to continue to make the telephone available as a modality of virtual care. The second clause pertains to investments for the longer term. Acknowledging that there are certain situations where video visits offer higher quality care than the telephone, steps must be taken to create systems in which video visits become much more accessible. However, realistically, this is a longer-term vision. Some of these steps are difficult and politically charged, related to enhancing the availability of high-speed Internet and making digital technologies available, including device access, affordable data plans, and enhanced digital literacy, to those who need them for health-related reasons. But if video visits are to become a mainstay of contemporary health care, such changes represent the only ways to ensure virtual care does not exacerbate health inequities into the future.

Finally, we are grateful to an anonymous reviewer of this manuscript for emphasizing the normative importance of cost in considerations of policy and funding for virtual care. We acknowledge that the telephone is widely available to the public and has the potential to drive increased use of health care resources given its widespread accessibility and ease of use. These observations raise the prospect of growing costs to health care systems specifically as a result of growth in the number of telephone visits. However, no clear evidence about the impacts of sustained funding of telephone visits on health system spending is yet available. The same is true of sustained funding and regular use of video visits. These will be important issues for continued research as sustained funding for the telephone as a modality of virtual care continues throughout and after the pandemic.

## Problem-driven planning

As planning for post-pandemic health care delivery continues, the focus should not be on technologies, but rather the problems we are trying to solve. This does not mean that there should be a stifling of innovation, but it does require careful thought as to why particular technologies are chosen and the inequities that may result. A thoughtful approach that enables telephone visits for appropriate uses and maintains investments in video visits and other emerging virtual care technologies for the longer term can ensure needs are met while striking a balance between access and quality of care.

Several publications have proposed directions for policy to promote health equity in virtual care during and beyond the pandemic^2,3,15–18^, and we do not plan to rehearse well-known strategies here. However, we emphasize the importance of three strategies that are most closely aligned with our argument. First, health system decision-makers should invest much more substantially in relationships with communities affected by health inequities, and seek out input on the value of virtual care and strategies to maintain access. Second, clinical leaders should work to develop clinical decision support tools that specify the clinical scenarios where a telephone is clinically appropriate, and where alternative modalities of care are required. Finally, governments must invest further in making high-speed Internet universally available and ensuring that people have access to the devices and data they need to engage in more advanced modalities of care into the future.

When the main user of the system, the patient, is at the center, it becomes clear that a single solution will not be sufficient for the variety of population needs. As providers and health systems plan for the more prominent use of virtual care into the future, it is imperative to ask whether our default solutions are accessible to everyone. If not, the digital divide will only continue to grow.

## Data Availability

Data sharing is not applicable to this article as no datasets were generated or analysed.
